# Postpartum Granulomatous Hypophysitis: A Case Study, Review of the Literature, and Discussion of Pathogenesis

**DOI:** 10.1155/2016/7510323

**Published:** 2016-08-24

**Authors:** Upasana Joneja, D. Craig Hooper, James J. Evans, Mark T. Curtis

**Affiliations:** ^1^Department of Pathology, Cell Biology and Anatomy, Thomas Jefferson University Hospitals, Philadelphia, PA, USA; ^2^Department of Cancer Biology, Thomas Jefferson University Hospitals, Philadelphia, PA, USA; ^3^Department of Neurosurgery, Thomas Jefferson University Hospitals, Philadelphia, PA, USA

## Abstract

Hypophysitis is a rare inflammatory condition of the pituitary gland that has three main histologic subtypes: lymphocytic hypophysitis (LH), granulomatous hypophysitis (GH), and xanthomatous hypophysitis (XH). Among these, LH is the most common and is strongly associated with the postpartum state, while XH is the least common. Many hypophysitis cases have been reported in the literature but only a few cases of postpartum GH have been discussed. Here, we describe a case of GH in a 24-year-old female presenting eleven days postpartum. We also review the current literature on postpartum GH and discuss the possible alterations in the immune environment during and after pregnancy that could explain this phenomenon. With more cases of GH being reported, the commonalities of female predominance, postpartum time of presentation, and occasional spontaneous resolution between LH and GH lend support to the theory that these two diseases likely represent spectrums of a single immunologic disorder.

## 1. Introduction

Hypophysitis, inflammation of the pituitary gland, commonly presents as a sellar mass on radiologic imaging and is often mistaken for a pituitary adenoma [1, 2]. It is rare and accounts for less than 1% of all sellar lesions identified on imaging [2]. The three main histologic subtypes of hypophysitis are lymphocytic hypophysitis (LH), granulomatous hypophysitis (GH), and xanthomatous hypophysitis (XH). Xanthogranulomatous and necrotizing hypophysitis are also considered subtypes of hypophysitis, but they are infrequently reported [3]. GH can occur as a primary process or as a consequence of preexisting systemic disease. Eighty-five cases of GH have been reported in the literature with 5 cases presenting during the postpartum state. The current report describes a case of postpartum GH and addresses the possibility that alterations in the immune environment associated with the antepartum and postpartum periods play a role in the development of GH.

## 2. Case Presentation

**Figure 1:**
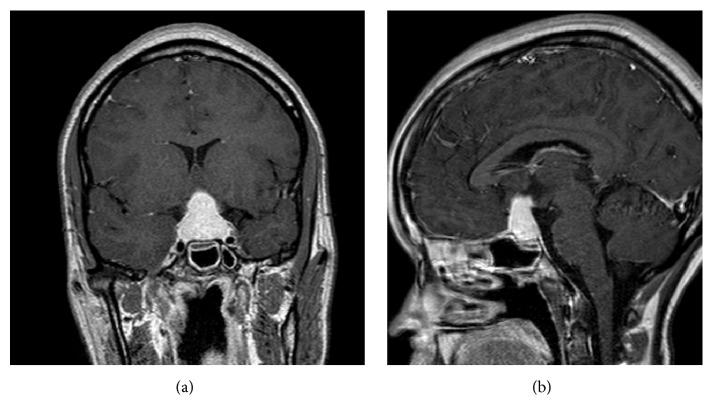
(a) 2.4 × 2.2 cm enhancing sellar lesion, coronal section. (b) A sagittal section of the same sellar mass with a 1.5 cm tertiary dimension.

**Figure 2:**
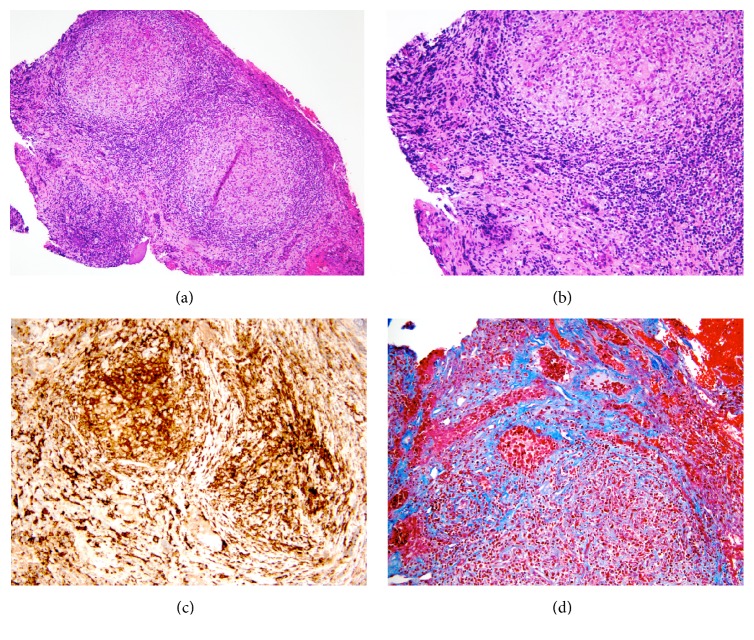
(a) A low magnification view of the sellar region showing multiple granulomas composed of histiocytes surrounded by lymphoplasmacystic infiltrates, 100x. (b) A higher magnification view of the granulomatous inflammation, 400x. (c) CD163 immunohistochemical stain highlighting the histiocytes within the granulomas, 400x. (d) Trichrome histochemical stain highlighting background fibrosis in blue, 400x.

A 24-year-old African American female G5P0023 presented to the emergency room with complaints of progressive, throbbing headaches and nausea for nine days. Eleven days earlier, she delivered a healthy full term baby via uncomplicated vaginal delivery. The patient also complained of blurry vision, fevers, lethargy, and photophobia. She denied any past medical history of migraines, seizures, eclampsia, or preeclampsia during pregnancy. The patient was febrile to 101.7°F and normotensive. An MRI revealed a 2.4 × 2.2 × 1.5 cm homogeneously enhancing sellar mass with a dark band surrounding the gland with infiltration into the base of the brain. Chiasmal compression, edema of the optic tracts, and mild dural enhancement anterior to the mass were noted. Pituitary stalk was difficult to visualize and its involvement could not be assessed. The MRI displayed features suggestive of an inflammatory process; however, a pituitary macroadenoma could not be completely ruled out (Figures 1(a) and 1(b)). In order to assess her pituitary function, a pituitary hormone panel was ordered and the results are shown in Table 1. The only major disturbance in the pituitary axis was low levels of ACTH and cortisol. The patient was placed on high dose of steroids. The options for management were discussed at length with the patient and the family including conservative management with observation versus surgery. Due to the symptoms of visual alterations, lethargy, and endocrine abnormalities, a decision to proceed with surgery was made. A partial resection of the sellar mass was performed and the pathologic diagnosis of the lesional tissue was granulomatous hypophysitis. No pituitary adenoma was identified. No Langerhans cells were noted. The specimen contained multiple well-formed granulomas situated within a background of markedly fibrotic stroma and residual adenohypophysis. The granulomas contained abundant CD163 positive macrophages and lymphocytes. Trichrome histochemical stain highlighted the increased fibrosis in the background (Figures 2(a)–2(d)). The sample was negative for fungal organisms on PAS and GMS stains, and no foreign material was identified. AFB stain and AFB immunofluorescence stain were negative, and no spirochetes were identified on Warthin-Starry stain. Aerobic, anaerobic, fungal, and AFB microbiologic cultures were also negative and vasculitis was not observed. No IgG4 positive plasma cells were identified on immunohistochemistry for IgG4, ruling out IgG4 related disease. Additional tests were performed to rule out other possible causes of GH. The patient did not have a history of sarcoid and angiotensin converting enzyme (ACE) levels were within normal limits. Immunologic markers were tested, including ANA panel, cytoplasmic ANCA, perinuclear ANCA, cryoglobulin, anti-Smith antibody, anti-smooth muscle/RNP antibody, anti-SSA, SSB antibodies, anti-PR3, antimyeloperoxidase, anti-CCP antibodies, and complements C3 and C4. The results of all of these tests were within normal ranges. Test for syphilis antibody was also negative. The patient did not have a history or symptoms of Crohn's disease. With no definitive systemic cause of the granulomatous inflammation identified, a final diagnosis of idiopathic granulomatous hypophysitis (IGH) was rendered. The patient had an uneventful postoperative course, and, on postoperative day 2, the patient reported no headaches or altered vision. The patient was discharged to home on steroids on postoperative day 4. The patient was seen as an outpatient 15 days postoperatively. At this time, she was in good state of health and denied complaints of headaches, blurry vision, or lethargy. No additional steroids were prescribed at this visit. Even though postoperative endocrinology labs and MRI of the brain were recommended, the patient was noncompliant and lost to follow-up for 1 year. During the 1-year postoperative follow-up, the patient was still symptom-free and denied taking any medications including hormonal therapy.

## 3. Discussion

Hypophysitis is a rare disorder with an estimated incidence of 1 case per 10 million people per year [1]. Of the main three histologic subtypes, LH is the most common, and GH is the next most common with XH being the least common. Disorders leading to GH as a consequence of systemic disease include sarcoidosis, Crohn's disease, pyoderma gangrenosum, syphilis, tuberculosis, pituitary adenomas, Langerhans cell histiocytosis, granulomatosis with polyangiitis, and foreign body granulomas secondary to Rathke's cleft cyst rupture. All these disorders need to be ruled out, as they were in this case prior to making a diagnosis of idiopathic granulomatous hypophysitis (IGH). Regarding radiologic work-up of pituitary disorders, certain MRI features can suggest an inflammatory process over pituitary adenoma. These include symmetric pituitary gland enlargement, thickened pituitary stalk, dark band surrounding the pituitary, marked and homogenous enhancement of the mass (not commonly associated with pituitary adenomas), and infiltration of adjacent structures [7]. In this case, the latter 3 features were present.

The only systemic review of IGH was by Hunn et al. in 2014 in which 82 cases were examined. In the reviewed cases, IGH cases predominantly affected females and had clinical symptoms of fever, nausea, vomiting, and histologic evidence of necrosis, all features seen in our case. In the review, these features were correlated with early presentation of the disorder [5]. Although the correlation between postpartum state and LH has been described, no such association has been proposed for IGH [3]. During our search of the literature, we discovered 5 cases of postpartum IGH (Table 2) [4, 6, 8]. There has been discussion that LH and GH may represent a spectrum of the same disorder with earlier stages showing mainly lymphoplasmacytic infiltrates and later stages progressing to giant cells and granulomatous inflammation [5, 9]. Some researchers suggest that IGH is a distinct entity because it lacks features of LH such as female preponderance, association with pregnancy, occasional spontaneous resolution, and association with autoimmune diseases [10]. However, as more cases of IGH are accruing, female predominance, the postpartum state, and occasional spontaneous resolution are starting to be recognized as features of the disease, lending support to the concept that LH and IGH may represent spectrums of the same disease.

Autoantibodies specific for pituitary autoantigens have been found in cases of both LH and IGH. Lupi et al. studied 28 cases of “autoimmune” hypophysitis which included cases of LH and IGH. They discussed five known prospective pathogenic autoantigens, growth hormone, alpha-enolase, pituitary gland-specific factors 1a and 2, secretogranin II, and two novel autoantigens, C14orf166 and chorionic somatomammotropin [11]. While pituitary autoantibodies have unknown clinical significance and can be detected in unrelated disorders, their presence in both LH and IGH suggests that these two conditions may have a common autoimmune pathogenesis. The identification of abundant macrophages bearing the M2-marker CD163 and abundant fibrosis in this case raises the possibility that postpartum IGH may involve a T helper 2 (Th2) response to autoantigens in the hypophysis. Prolactin, the levels of which are elevated in the postpartum period, has immunostimulatory effects and can promote autoimmunity [12]. It has been proposed that, in Th2-mediated autoimmune processes, prolactin may be involved in the progression of Th2-mediated autoimmunity through its direct effects on Th2-related antibody production [13, 14]. It is possible that postpartum LH and GH share pathogenic mechanisms.

Immune system changes associated with pregnancy may play a role in IGH as they are known to do in LH. Pregnancy is a complex immunologic state in which a bias towards Th2 protects the fetus [15]. The decrease in Th1 cytokines and increase in Th2 cytokines during pregnancy are well documented [16]. Whereas an enhanced Th2/Th1 ratio seems necessary for fetal protection, a Th2 type cytokine profile also favors antibody synthesis [17]. It has been demonstrated* in vitro* that IL-10 induction of a Th2 response significantly augments antibody production [18]. The predominantly Th2 immune state of pregnancy that creates an immune environment favorable to the fetus undergoes significant changes in the postpartum period [19]. A relationship between pregnancy and autoimmunity is well documented and has been shown to encompass numerous autoimmune diseases including postpartum thyroiditis, Sheehan's syndrome, and peripartum cardiomyopathy [20]. The prevailing theory is that these postpartum autoimmune disorders result in part from immune system changes including alterations of cytokines and autoantibody levels that occur in the postpartum period when there is a transition from the temporary immune tolerance/immune suppression of pregnancy to an immune environment approaching that of the prepregnancy state [21]. During this postpartum transition, a rebound or overcompensation of the immune system may occur including elevation of levels of proinflammatory cytokines and pathogenic actions of autoantibodies. Such events may play a role in postpartum hypophysitis. In some cases of postpartum hypophysitis, these physiologic disturbances are temporary and limited to the postpartum period, as was the case in 2 of 5 postpartum cases of IGH discussed in Table 2 [4, 8]. It can be speculated that the autoantibodies produced in the Th2 environment of pregnancy may play a role in the development of autoimmunity leading to IGH in the postpartum state. The exact role that M2 macrophages play in postpartum IGH is not known. Further studies are needed to better characterize the multiple aspects of the postpartum immune environment including macrophage polarization, cytokine levels, and pituitary autoantibody production in these patients.

## 4. Conclusion

GH is a rare entity that is most often mistaken for pituitary adenoma prior to biopsy. Other systemic disease processes that can cause GH must be ruled out prior to a definitive diagnosis of IGH since the clinical manifestations of IGH often totally resolve following debulking surgery. IGH can present during the postpartum period and may share a pathogenic mechanism with postpartum LH. While the etiology of LH and IGH remains unclear, the immune environment of pregnancy and of the postpartum period may play a role in a subset of these disorders. Documentation and further analysis of such cases are needed as this will enhance our understanding of this disease and increase awareness of IGH among clinicians.

## Figures and Tables

**Table 1:** Results of the pituitary axis hormone levels of the patient.

Hormone	Patient's level	Reference range
Adrenocorticotropic hormone (ACTH)	<9	9–46 pg/mL

Cortisol	AM: 1.6 PM: 1.4	AM: 16–20 mcg/dL PM: 2–12 mcg/dL

Luteinizing hormone	0.1	Follicular phase: 2.4–12.6 mIU/mL Ovulation phase: 14–95.6 mIU/mL Luteal phase: 1–11.4 mIU/mL

Follicle stimulating hormone (FSH)	3.7	Follicular phase: 3.5–12.5 mIU/mL Ovulation phase: 4.7–21.5 mIU/mL Luteal phase: 1.7–7.7 mIU/mL

Prolactin	19	0–29 ng/mL

Thyroid stimulating hormone (TSH)	1.31	0.3–5.0 mIU/mL

Human growth hormone	1.6	<10 ng/mL

**Table 2:** Documented cases of postpartum idiopathic granulomatous hypophysitis with patient demographics and follow-up data.

Publication	Number of cases	Demographics	Pregnancy status	Treatment	Follow-up
Tashiro et al. 2002 [6]	3	24, 31, 33 F	Postpartum, postpartum, pregnant	Treatment not discussed in the study	No follow-up available

Mehndiratta et al. 2007 [4]	1	29	Pregnant (6 months)	Transsphenoidal decompression and hormone replacement	Patient required hormone replacement for approximately 10 weeks and then was symptom-free

Demetri et al. 2010 [8]	1	29 F	Postpartum (2 weeks)	Transsphenoidal subtotal resection with desmopressin and thyroxine therapy following surgery	Spontaneous resolution of residual mass within 5 months

## References

[1] Thodou E., Asa S. L., Kontogeorgos G., Kovacs K., Horvath E., Ezzat S. (1995). Lymphocytic hypophysitis: clinicopathological findings. *Journal of Clinical Endocrinology and Metabolism*.

[2] Cheung C. C., Ezzat S., Smyth H. S., Asa S. L. (2013). The spectrum and significance of primary hypophysitis. *Journal of Clinical Endocrinology and Metabolism*.

[3] Bellastella A., Bizzarro A., Coronella C., Bellastella G., Sinisi A. A., De Bellis A. (2003). Lymphocytic hypophysitis: a rare or underestimated disease?. *European Journal of Endocrinology*.

[4] Mehndiratta M. M., Phul P., Singh A. K., Garg S., Bali R. (2007). Granulomatous hypophysitis—an interesting and rare causes mimicking pituitary mass. *Journal of Associations of Physicians of India*.

[5] Hunn B. H. M., Martin W. G., Simpson S., Mclean C. A. (2014). Idiopathic granulomatous hypophysitis: a systematic review of 82 cases in the literature. *Pituitary*.

[6] Tashiro T., Sano T., Xu B. (2002). Spectrum of different types of hypophysitis: a clinicopathologic study of hypophysitis in 31 cases. *Endocrine Pathology*.

[7] Lury K. M. (2005). Inflammatory and infectious processes involving the pituitary gland. *Topics in Magnetic Resonance Imaging*.

[8] Demetri C., Shoukri K. C., Taylor S. L., Silva J. E. (2010). Postpartum granulomatous hypophysitis with sphenoid sinus involvement: a case study. *Endocrine Practice*.

[9] Ludmerer K., Kissane J. (1984). Primary hypothyroidism and hypopituitarism in a young woman. *The American Journal of Medicine*.

[10] Caturegli P., Newschaffer C., Olivi A., Pomper M. G., Burger P. C., Rose N. R. (2005). Autoimmune hypophysitis. *Endocrine Reviews*.

[11] Lupi I., Broman K. W., Tzou S.-C., Gutenberg A., Martino E., Caturegli P. (2008). Novel autoantigens in autoimmune hypophysitis. *Clinical Endocrinology*.

[12] Orbach H., Shoenfeld Y. (2007). Hyperprolactinemia and autoimmune diseases. *Autoimmunity Reviews*.

[13] De Bellis A., Bizzarro A., Pivonello R., Lombardi G., Bellastella A. (2005). Prolactin and autoimmunity. *Pituitary*.

[14] De Bellis A., Ruocco G., Battaglia M. (2008). Immunological and clinical aspects of lymphocytic hypophysitis. *Clinical Science*.

[15] Sykes L., MacIntyre D. A., Yap X. J., Ponnampalam S., Teoh T. G., Bennett P. R. (2012). Changes in the Th1 : Th2 cytokine bias in pregnancy and the effects of the anti-inflammatory cyclopentenone prostaglandin 15-deoxy-Δ ^12,14^-prostaglandin J_2_. *Mediators of Inflammation*.

[16] Reinhard G., Noll A., Schlebusch H., Mallmann P., Ruecker A. V. (1998). Shifts in the TH1/TH2 balance during human pregnancy correlate with apoptotic changes. *Biochemical and Biophysical Research Communications*.

[17] Margni R. A., Zenclussen A. C. (2001). During pregnancy, in the context of a Th2-type cytokine profile, serum IL-6 levels might condition the quality of the synthesized antibodies. *American Journal of Reproductive Immunology*.

[18] Xu Q., Katakura Y., Yamashita M. (2004). IL-10 augments antibody production in in-vitro immunized lymphocytes by inducing a Th2-type response and B cell maturation. *Bioscience, Biotechnology and Biochemistry*.

[19] Muller A. F., Drexhage H. A., Berghout A. (2001). Postpartum thyroiditis and autoimmune thyroiditis in women of childbearing age: recent insights and consequences for antenatal and postnatal care. *Endocrine Reviews*.

[20] Buyon J. P. (1998). The effects of pregnancy on autoimmune diseases. *Journal of Leukocyte Biology*.

[21] Singh N., Perfect J. R. (2007). Immune reconstitution syndrome and exacerbation of infections after pregnancy. *Clinical Infectious Diseases*.

